# *cpt1b* Regulates Cardiomyocyte Proliferation Through Modulation of Glutamine Synthetase in Zebrafish

**DOI:** 10.3390/jcdd11110344

**Published:** 2024-11-01

**Authors:** Xiaohan Cheng, Jingyi Ju, Wenping Huang, Zongyi Duan, Yanchao Han

**Affiliations:** Institute for Cardiovascular Science and Department of Cardiovascular Surgery of the First Affiliated Hospital of Soochow University, Suzhou Medical College, Soochow University, Suzhou 215000, China

**Keywords:** cardiomyocyte proliferation, *cpt1b*, *glul*, metabolism, zebrafish

## Abstract

Carnitine palmitoyltransferase 1b (Cpt1b) is a crucial rate-limiting enzyme in fatty acid metabolism, but its role and mechanism in early cardiac development remains unclear. Here, we show that *cpt1b* regulates cardiomyocyte proliferation during zebrafish development. Knocking out entire *cpt1b* coding sequences leads to impaired cardiomyocyte proliferation, while cardiomyocyte-specific overexpression of *cpt1b* promotes cardiomyocyte proliferation. RNA sequencing analysis and pharmacological studies identified glutamine synthetase as a key downstream effector of *cpt1b* in regulating cardiomyocyte proliferation. Our study elucidates a novel mechanism whereby *cpt1b* promotes zebrafish cardiomyocyte proliferation through glutamine synthetase, which provides new perspectives on the significance of fatty acid metabolism in heart development and the interplay between fatty acid and amino acid metabolic pathways.

## 1. Introduction

Cardiomyocyte proliferation is essential for heart development and regeneration. In the zebrafish embryos, cardiac progenitor cells converge at the midline and subsequently undergo asymmetric movements, culminating in the formation of a tubular heart, which then undergoes cardiac looping and jogging movements to give rise to the distinct heart chambers [1,2,3]. Concurrent with the widespread migration and morphogenesis, massive new cardiomyocytes are generated from progenitor cells and/or existing cardiomyocytes, facilitating the rapid growth and expansion of the heart chambers [4,5].

The regulation of cardiomyocyte proliferation is a complex process involving multiple signaling pathways and molecular mechanisms. Recent studies have highlighted the importance of metabolic regulation in cardiomyocyte proliferation [6,7,8,9]. Fatty acid metabolism, particularly the β-oxidation pathway, plays a critical role in energy production and cellular growth during development and tissue proliferation. Carnitine palmitoyltransferase 1 (CPT1), a rate-limiting enzyme instrumental in facilitating fatty acid transport into the mitochondria, thereby fueling β-oxidation, is a crucial regulator of this process [10]. The CPT1 family comprises three major tissue-specific isoforms: CPT1A, CPT1B, and CPT1C [11,12,13,14], among which CPT1B is the most abundant in the heart and skeletal muscle [15,16,17]. In neonatal and adult mouse hearts, inhibition of Cpt1b has been shown to stimulate the proliferation of cardiomyocytes, while concurrently impeding their maturation [18,19]. However, a recent study has unveiled that fatty acid oxidation and *cpt1b* play an indispensable role in cardiomyocyte proliferation during zebrafish ventricle regeneration [20]. Furthermore, knocking out *cpt1b* in zebrafish has been found to augment glucose utilization and protein deposition in the liver and muscle [21]. Despite these findings, the precise role of *cpt1b* in early heart development and cardiomyocyte proliferation remains obscure, necessitating further investigation to elucidate its mechanisms of action.

Glutamine, a non-essential amino acid, serves as a critical precursor for biosynthesis and energy production, playing a pivotal role in maintaining tissue growth and homeostasis [22,23,24]. The enzymatic synthesis of glutamine within cells is catalyzed by glutamine synthetase (GS), also known as glutamate-ammonia ligase (GLUL) [25]. Comprehensive studies have shown that GLUL can modulate cancer cell proliferation and tumor growth under diverse conditions, either dependent on or independent of glutamine synthesis and metabolism [26,27,28]. However, the function of GLUL in cardiac development and cardiomyocyte proliferation is still largely unknown.

In this study, we generated a *cpt1b* knockout and a cardiac-specific *cpt1b* overexpressing transgenic zebrafish line to elucidate the impact of *cpt1b* on cardiomyocyte proliferation in zebrafish embryos. We further employed RNA sequencing and pharmacological approaches to unravel the role of glutamine biosynthesis and Glul genes in this process.

## 2. Materials and Methods

### 2.1. Zebrafish Husbandry

Wild-type and transgenic zebrafish of the AB strain were used in this study. They were maintained in a dedicated zebrafish breeding unit (ESEN Science & Technology, Beijing, China) with a daily light cycle of 14 h of light and 10 h of darkness. The zebrafish were fed with Artemia twice daily. The water temperature was approximately 28 °C, with a pH of around 7.2, and conductivity of approximately 500 µS/cm. Embryos were obtained through natural mating and cultured at 28.5 °C. The knock-in, transgenic and knockout strains used in this study were *pcna^mGFP^*^,*pd392*^ [29], *Tg(myl7:H2A-mCherry)^sd12^* [30], and *Tg(myl7:cpt1b-TBFP)^hs3^* (this study) and *cpt1b^hs4^* (this study).

### 2.2. Generation of cpt1b Knockout Zebrafish

The *cpt1b* knockout allele was generated using CRISPR/Cas9-mediated genomic editing technology. Briefly, guide RNAs targeting the 5’ UTR (5′-TGCTTTAAGCCTGTATAATGG-3′) and 3’ UTR (5′-TGAAGCTTCTTGGTTTACGG-3′) of the *cpt1b* gene were generated and co-injected with Cas9 protein into one-cell stage zebrafish embryos as described [31]. Injected embryos were raised to adulthood and screened for germline transmission with PCR, followed by Sanger sequencing of amplicons from F1 animals to confirm the mutation. The *cpt1b* knockout used in this study was genotyped with the following primers which can produce amplicons of 398 bp or 278 bp for wild-type or knockout alleles, respectively:

*cpt1b-1f*: 5′-AGATCTTTCCACTGTTGCCATT-3′;

*cpt1b-2f*: 5′-TGCAGCAACATCAACGCAGTC-3′; 

*cpt1b-2r*: 5′-ATTCGCTAGGCTTGTTACTTGC-3′.

### 2.3. Generation of Tg(myl7:cpt1b-TBFP) Transgenic Zebrafish

To generate the *Tg(myl7:cpt1b-TBFP)* transgenic line, the 2.7 kb *cpt1b* coding sequences were amplified from a zebrafish cDNA library and inserted into a Tol2/I-SceI vector containing a 0.9 kb *myl7* promoter sequence and a P2A-TBFP coding sequence using Golden Gate cloning [31]. The plasmid was then purified, linearized with I-SceI meganuclease (R0694S, New England BioLabs, Ipswich, MA, USA), and microinjected into one-cell stage zebrafish embryos. Stable transgenic founders were identified by screening zebrafish embryos that exhibited cardiac blue fluorescence under a Zeiss Axio Zoom V16 stereomicroscope (Oberkochen, Germany).

### 2.4. In Situ Hybridization 

To generate the *cpt1b* probe template, a partial *cpt1b* coding sequence was amplified from a zebrafish cDNA library with primers *cpt1b* fwd GAAGCACATGGATACGATCC, rev GTGTCAATGAGATTGAGCTG. The resulting amplicon was cloned into a customized vector and subsequently used as a template to transcribe a Digoxigenin-labeled RNA probe in vitro. In situ hybridization and imaging were performed as previously described [32].

### 2.5. Cryosection

Adult zebrafish were anesthetized with a 0.2 mg/mL tricaine solution (T0941-25G, Tokyo Chemical Industry, Tokyo, Japan), and the hearts were excised and fixed in 4% paraformaldehyde (PFA, P6148, Sigma-Aldrich, St. Louis, MO, USA) overnight at 4 °C. Subsequently, the hearts were immersed in a 30% sucrose solution (A502792-0500, Sangon Biotech, Shanghai, China) and embedded in an OCT (optimal cutting temperature) compound (4583, Sakura Finetek, Tokyo, Japan). The heart blocks were mounted on a cryostat and sectioned into 10 µm thick slices with a microtome (CM1950, Leica Biosystems, Wetzlar, Germany) and the slides were dried on a hot surface at 42 °C for a duration of 2 h.

### 2.6. Drug Treatment

L-methionine sulfoximide (MSO, M5379, Sigma-Aldrich, St. Louis, MO, USA) was dissolved in reverse osmosis water to a final concentration of 250 mM and stored at −20 °C. Zebrafish embryos were screened based on fluorescence expression and subsequently treated with egg water either with or without 250 µM MSO at 3 dpf (days post fertilization). Twenty-four hours later, the embryos were rinsed and fixed with 4% PFA overnight at 4 °C.

### 2.7. Cardiomyocyte Proliferation Assay

Embryonic zebrafish hearts were carefully excised using fine-tip tweezers and subsequently mounted on the bottom of glass-bottomed Petri dishes containing 0.8% low-melting-point agarose (A600015, Sangon Biotech, Shanghai, China). Confocal microscopic images were acquired using a Zeiss LSM 880 inverted confocal microscope and Zen 3.1 software. Cardiomyocytes were quantified using ImageJ software (version 1.54f). The cardiomyocyte proliferation index was calculated by dividing the number of Pcna^+^/mCherry^+^ cells by the total number of mCherry^+^ cells.

### 2.8. Quantification of Adult Zebrafish Ventricular Area

Adult zebrafish were anesthetized using tricaine and the hearts were excised and briefly fixed in 4% PFA. Subsequently, the hearts were imaged under a Zeiss stereo microscope and ventricular areas were measured using Zen 3.1 software.

### 2.9. RNA Sequencing

Total RNA was extracted from a pool of ten adult zebrafish hearts for each biological replicate. Library preparation, RNA sequencing and bioinformatic analysis were performed at Gene Denovo Biotechnology (Guangzhou, China). Briefly, RNA quality was assessed on an Agilent 2100 Bioanalyzer (Agilent Technologies, Santa Clara, CA, USA). Then, the cDNA libraries were constructed using NEBNext Ultra RNA Library Prep Kit for Illumina (7530, New England Biolabs, Ipswich, MA, USA), and were sequenced using an Illumina Novaseq6000 platform (San Diego, CA, USA). Differential gene analysis was performed using DESeq2 software (version 1.24.0) [33] with |log2(fold change)| ≥ 1 and *p*-value < 0.05 serving as the criteria to identify differentially expressed genes.

### 2.10. Quantitative RT-PCR

Total RNA was extracted from 30 zebrafish embryos or 4–8 adult zebrafish hearts per biological replicate using Trizol (R0016, Beyotime Biotechnology, Shanghai, China). Subsequently, 0.5 μg total RNA was reversed transcribed using HiScript II Q RT SuperMix (R223-01, Vazyme, Nanjing, China) for qPCR. The primers used were listed as follows (5’-3’): 

*cpt1b*: fwd: CTCCCCAACAGCAGCTCAAT, rev: AAGCGGAAAGAATCCGTCTCA;

*glula*: fwd: CACTAAGGAAATGCGGGAAG, rev: AGTGAGTCGACGAGCATTGT;

*glulb*: fwd: CACCAACTTCAGCACCAAGG, rev: TTGTCCAGCCCTCCTTTAGG;

*rpl4*: fwd: GGCCAGGGTGCTTTCGGAAA, rev: TTGGCCATGACAAGGGATGG.

qPCR was performed using AceQ Universal SYBR qPCR Master Mix (Q511-02, Vazyme, China) on an ABI StepOnePlus Real-Time PCR System. Gene expression was quantified using the ΔΔCT method with *rpl4* as an internal control. At least three independent biological replicates were performed for each experiment.

### 2.11. Statistical Analysis

All data are presented as mean ± standard deviation (SD). Statistical analyses were performed using Student’s *t*-test and/or one-way analysis of variance (ANOVA) unless otherwise stated.

## 3. Results

### 3.1. Loss of cpt1b Function Inhibits Cardiomyocyte Proliferation in Zebrafish Embryos

**Figure 1:**
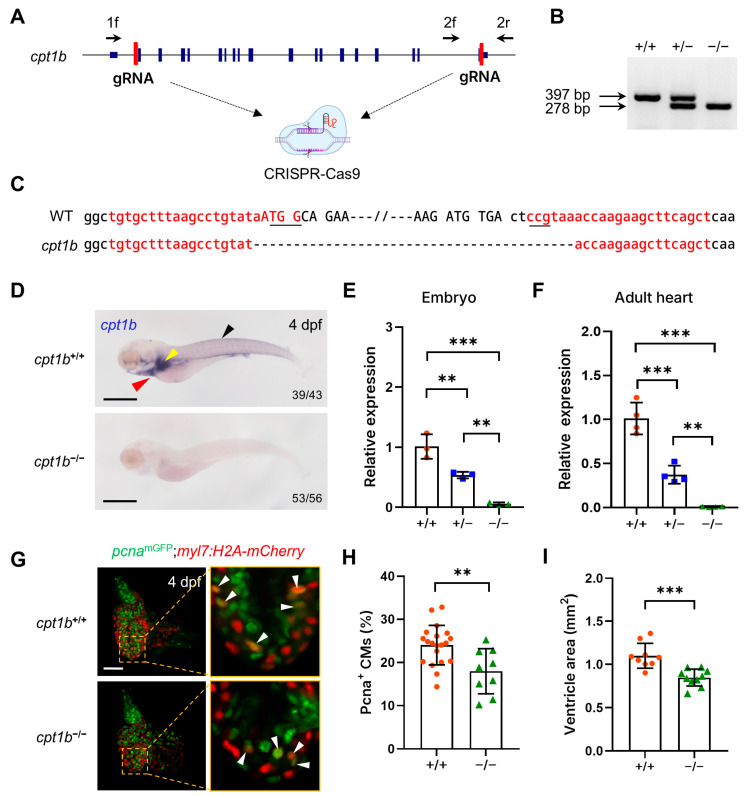
Loss of *cpt1b* function inhibits cardiomyocyte proliferation in zebrafish embryos. (**A**) A schematic representation of the design for generating the *cpt1b* knockout zebrafish. (**B**) A representative agarose gel electrophoresis image for the genotyping of *cpt1b*^+/+^, *cpt1b*^+/−^, and *cpt1b*^−/−^ zebrafish. (**C**) Genomic sequences of wild-type (WT) and *cpt1b* knockout zebrafish around *cpt1b* guide RNA target sites. The guide RNA target sequences are denoted by red letters, with the protospacer adjacent motif (PAM) sequences underscored, and the coding sequences are represented by capital letters. (**D**) Whole-mount in situ hybridization analysis of *cpt1b* expression in *cpt1b*^+/+^ and *cpt1*^−/−^ zebrafish embryos at 4 dpf. The black triangle indicates muscle, the yellow triangle indicates liver, and the red triangle indicates heart. Scale bar: 500 μm. (**E**) Quantitative RT-PCR analysis of *cpt1b* mRNA expression in 4 dpf *cpt1b*^+/+^, *cpt1b*^+/−^, and *cpt1b*^−/−^ embryos. (**F**) Quantitative RT-PCR analysis of *cpt1b* mRNA expression in the hearts of adult *cpt1b*^+/+^, *cpt1b*^+/−^, and *cpt1b*^−/−^ zebrafish. ** *p* < 0.01, *** *p* < 0.001. (**G**) Representative maximum intensity projection confocal images of dissected hearts from 4 dpf *Tg*(*pcna^mGFP^;myl7:H2A-mCherry;cpt1b^+/+^*) and *Tg*(*pcna^mGFP^;myl7:H2A-mCherry;cpt1b*^−/−^) zebrafish. Enlarged single optical section images of the boxed regions are shown on the right. White triangles indicate proliferating cardiomyocytes. Scale bar: 50 µm. (**H**) Quantitative analysis of proliferating cardiomyocytes in G. *n* = 9–20. ** *p* < 0.01. (**I**) Quantification of ventricular areas of adult *cpt1b*^+/+^ and *cpt1b*^−/−^ zebrafish. *n* = 9–10. *** *p* < 0.001.

To circumvent the potential genetic compensation and/or cryptic downstream translational initiation events [34,35], we utilized the CRISPR/Cas9 genomic editing system to generate a complete knockout of the coding region of the *cpt1b* gene (Figure 1A). Since the two guide RNAs target the 5’ untranslated region (UTR) and 3’ UTR, respectively, the resultant knockout lacks the entire *cpt1b* coding sequences (Figure 1B,C). Consistent with the design, when detected with in situ hybridization using a riboprobe spanning exons 10 to 16 of *cpt1b* gene, *cpt1b* expression was detected in the heart, liver, and muscle tissues of wild-type (WT) embryos but were completely absent in the knockout embryos (Figure 1D). Subsequently, we quantified the expression level of *cpt1b* coding sequences using reverse transcription quantitative polymerase chain reaction (RT-qPCR). As shown in Figure 1E, the expression level of *cpt1b* coding sequences in heterozygous embryos (*cpt1b*^+/−^) was significantly downregulated, reaching 47% of that in wild-type embryos, whereas the expression of *cpt1b* in homozygous embryos (*cpt1b*^−/−^) was virtually abolished, plummeting to a mere 0.06% of the wild-type level. Similarly, in adult hearts, the expression levels of *cpt1b* coding sequences of *cpt1b*^+/−^ and *cpt1b*^−/−^ zebrafish were also markedly decreased to 37% and 0.01% of the wild-type level, respectively (Figure 1F). These data collectively demonstrate that the *cpt1b* knockout constitutes a null mutation, devoid of functional coding sequences, thereby providing a valuable tool for investigating the consequences of *cpt1b* loss in subsequent studies.

To investigate the effects of *cpt1b* deficiency on cardiomyocyte proliferation during zebrafish development, we crossed the *cpt1b* knockout zebrafish with a double-transgenic zebrafish line, *Tg(pcna*^mGFP^;*myl7:H2A-mCherry)*, which features a cardiac-specific *myl7* promoter-driven expression of nuclear H2A-mCherry fusion protein in cardiomyocytes. Additionally, this line harbors a monomeric green fluorescent protein (mGFP) knock-in at the C-terminal of the *proliferating cell nuclear antigen (pcna)* gene, resulting in a fusion of mGFP to the endogenous Pcna protein to label the nuclei of cycling cells with green fluorescence. Consequently, the dual-fluorescence labeling enables the real-time, in vivo monitoring of cardiomyocyte proliferation (Figure 1G). Subsequently, the hearts were excised at 4 dpf and imaged under confocal laser microscope. In the hearts of wild-type (*cpt1b*^+/+^) zebrafish, approximately 24% of cardiomyocytes were Pcna-positive, whereas in the hearts of *cpt1b*^−/−^ zebrafish embryos, only 18% of cardiomyocytes were Pcna-positive (Figure 1G,H). Consequently, the ventricles of adult *cpt1b* knockout zebrafish exhibited a marked 23% reduction in size compared to those of their wild-type siblings (Figure 1I). These data suggest that the *cpt1b* mutation exerts a significant inhibitory effect on cardiomyocyte proliferation during zebrafish heart development.

### 3.2. Overexpression of cpt1b Promotes Cardiomyocyte Proliferation in Zebrafish Embryos

**Figure 2:**
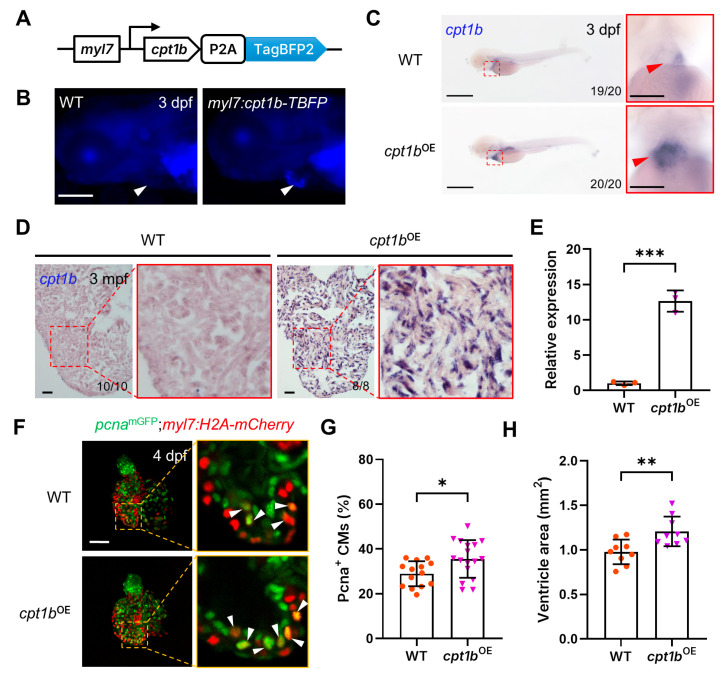
Overexpression of *cpt1b* promotes cardiomyocyte proliferation in zebrafish embryos. (**A**) A schematic representation of the *Tg(myl7:cpt1b-TBFP)* construct. (**B**) A stereo fluorescence image of a 3 dpf wild-type sibling and *Tg(myl7:cpt1b-TBFP)* embryos. The triangles indicate the embryonic hearts. Scale bar: 200 µm. (**C**) Whole-mount in situ hybridization analysis of *cpt1b* expression with WT and *cpt1b*^OE^ zebrafish embryos at 3 dpf. The red boxes demarcate enlarged ventral views of the hearts (red triangles). Scale bar: 500 µm. (**D**) In situ hybridization analysis of *cpt1b* expression in cryosections of adult WT and *cpt1b*^OE^ zebrafish hearts. The red boxes show enlarged views of the denoted regions. Scale bar: 50 µm. (**E**) Quantitative RT-PCR analysis of *cpt1b* expression in the hearts of adult WT and *cpt1b*^OE^ zebrafish. *** *p* < 0.001. (**F**) Representative maximum intensity projection confocal images of dissected hearts from 3 dpf *Tg(pcna^mGFP^;myl7:H2A-mCherry)* and *Tg(pcna^mGFP^;myl7:H2A-mCherry;myl7:cpt1b-TBFP)* embryos. Enlarged single optical section images of the boxed regions are shown on the right. White triangles indicate proliferating cardiomyocytes. Scale bar: 50 µm. (**G**) Quantitative analysis of proliferating cardiomyocytes in F. * *p* < 0.05. *n* = 14–17. (**H**) Quantification of ventricular areas of adult wild-type and *cpt1b*^OE^ zebrafish. *n* = 9–10. ** *p* < 0.01.

To investigate the impact of elevated *cpt1b* expression on cardiomyocyte proliferation, we generated a transgenic zebrafish line, hereafter referred to as *Tg(myl7:cpt1b-TBFP)* or *cpt1b*^OE^ for short, which overexpresses *cpt1b* specifically in cardiomyocytes by employing the cardiac-specific *myl7* promoter to drive the expression of the zebrafish *cpt1b* gene and mTagBFP2, a monomeric blue fluorescent protein, separated by the self-cleaving peptide P2A (Figure 2A), which can be screened by blue fluorescence in the heart (Figure 2B). Whole-mount in situ hybridization revealed that the expression of *cpt1b* was significantly increased in the hearts of *cpt1b*^OE^ embryos, without affecting its expression in other tissues and organs, compared to their wild-type siblings at 3 dpf (Figure 2C). Furthermore, the expression of the *cpt1b* gene was also significantly increased in the cardiomyocytes of adult *cpt1b*^OE^ zebrafish hearts (Figure 2D), exhibiting a remarkable 11-fold increase compared to their WT siblings (Figure 2E). These results demonstrate that *cpt1b* expression is significantly upregulated in the cardiomyocytes of *Tg(myl7:cpt1b-TBFP)* transgenic zebrafish.

We then crossed the *Tg(myl7:cpt1b-TBFP)* zebrafish with the *Tg(pcna^mGFP^;myl7:H2A-mCherry)* zebrafish to investigate the impact of *cpt1b* overexpression on cardiomyocyte proliferation. Confocal microscopic imaging and quantification revealed that the proportion of Pcna-positive cardiomyocytes in the *cpt1b*^OE^ hearts was significantly elevated, exhibiting a 20% increase compared to their WT sibling hearts (Figure 2F,G). Notably, the ventricular areas of adult *cpt1b*^OE^ zebrafish were increased by 27% compared to those of their wild-type counterparts (Figure 2H). Collectively, these findings suggest that cardiomyocyte-specific *cpt1b* overexpression can enhance cardiomyocyte proliferation during zebrafish heart development.

### 3.3. Glul Mediates cpt1b-Induced Cardiomyocyte Proliferation in Zebrafish

**Figure 3:**
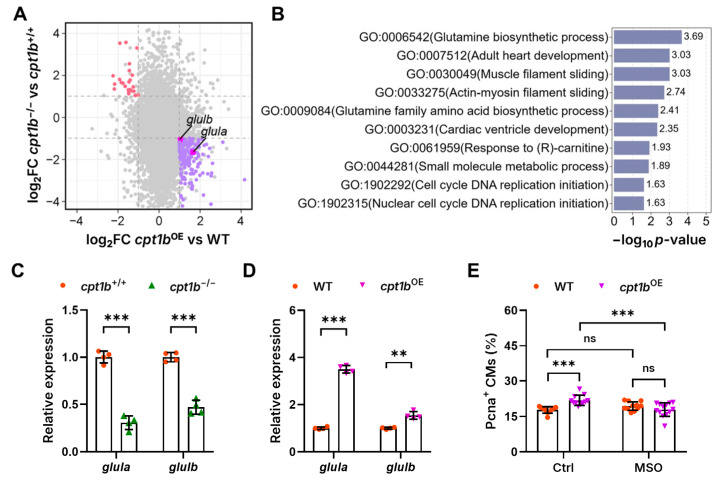
Transcriptomic analysis reveals the potential contribution of Glul in *cpt1b*-regulated cardiomyocyte proliferation in zebrafish. (**A**) Nine-quadrant plot illustrating the log2(fold change) of recovered genes in the *cpt1b*^−/−^ vs. *cpt1b*^+/+^ analysis and *cpt1b*^OE^ vs. WT analysis. Red dots denote genes that were significantly upregulated in *cpt1b*^−/−^ hearts and concomitantly downregulated in *cpt1b*^OE^ hearts, blue dots represent genes that were downregulated in *cpt1b*^−/−^ hearts and upregulated in *cpt1b*^OE^ hearts, and pink dots highlight the two glutamine synthetase genes. (**B**) Gene Ontology enrichment analysis of 154 differentially expressed genes revealed significant enrichment of biological processes. The *y*-axis represents the enriched biological processes, and the *x*-axis represents the negative logarithm of the *p*-value. (**C**) Quantitative RT-PCR analysis of *glula* and *glulb* expression in *cpt1b*^+/+^ and *cpt1b*^−/−^ hearts. (**D**) Quantitative RT-PCR analysis of *glula* and *glulb* expression in WT and *cpt1b*^OE^ hearts. (**E**) Quantitative analysis of Pcna-positive cardiomyocytes in 4 dpf WT and *cpt1b*^OE^ zebrafish embryos treated with either vehicle (Ctrl) or 250 µM MSO. *n* = 10–13. ** *p* < 0.01, *** *p* < 0.001, and ns indicates *p* > 0.05.

To decipher the transcriptomic adaptations in the hearts with modified *cpt1b* expression and to uncover the potential molecular mechanisms underlying *cpt1b*-regulated cardiomyocyte proliferation, we performed RNA sequencing on hearts isolated from adult *cpt1b*-knockout (*cpt1b*^−/−^) and *cpt1b*-overexpressing (*cpt1b*^OE^) zebrafish alongside their corresponding wild-type siblings. Primary analysis disclosed a total of 997 upregulated and 3245 downregulated genes in the hearts of *cpt1b*^−/−^ zebrafish, whereas 417 genes were upregulated and 129 genes were downregulated in the *cpt1b*^OE^ hearts. Subsequent conjoint analysis identified 171 genes that exhibited a reciprocal expression pattern, being upregulated in *cpt1b*^−/−^ hearts and downregulated in *cpt1b*^OE^ hearts, or vice versa (Figure 3A, Supplementary Table S1). 

Gene Ontology (GO) enrichment analysis revealed that these genes are predominantly implicated in biological processes such as glutamine biosynthesis, adult heart development, small molecule metabolism, cell cycle DNA replication, and myofilament sliding (Figure 3B). Notably, glutamine biosynthesis emerged as the most prominently enriched process. Given that the zebrafish genome possesses three glutamine synthesis genes, and two of them, *glula* and *glulb*, were upregulated in *cpt1b*^OE^ hearts and downregulated in *cpt1b*^−/−^ hearts (Figure 3A), we hypothesized that they may play a crucial role in mediating *cpt1b*-induced cardiomyocyte proliferation in zebrafish. To test this hypothesis, we conducted RT-qPCR to validate their expression in the hearts. In accordance with the RNA sequencing results, RT-qPCR analysis confirmed that the expression of *glula* and *glulb* was significantly downregulated, by 69% and 53%, respectively, in *cpt1b*^−/−^ hearts, whereas their expression was markedly increased, by 2.5-fold and 54%, respectively, in the *cpt1b*^OE^ hearts relative to their wild-type counterparts (Figure 3C,D). These data demonstrate that the expression of *glula* and *glulb* is indeed subject to regulation by *cpt1b* levels, suggesting a critical role for *cpt1b* in modulating the expression of glutamine synthetase genes, which may, in turn, influence cardiomyocyte proliferation.

L-methionine sulfoximine (MSO), a glutamate analog, irreversibly binds to the active site of glutamine synthetase, thereby inhibiting its enzymatic activity [36]. To further elucidate the potential role of Glul in regulating cardiomyocyte proliferation in zebrafish, we collected double- or triple-transgenic embryos from the cross between *Tg(myl7:cpt1b-TBFP)* and *Tg(pcna^mGFP^;myl7:H2A-mCherry)* zebrafish, and treated them either with or without 250 µM MSO at 3 dpf. After 24 h of treatment, the embryos were fixed and their hearts were carefully dissected and subsequently imaged using a confocal laser microscope to assess cardiomyocyte proliferation. In wild-type embryos lacking *cpt1b* overexpression, the proportion of Pcna-positive cardiomyocytes in MSO-treated hearts was statistically indistinguishable from that observed in vehicle-treated hearts. In contrast, the proportion of Pcna-positive cardiomyocytes in *cpt1b*^OE^ embryos without MSO treatment exhibited a marked 23% increase compared to wild-type hearts. However, MSO treatment dramatically reduced the proportion of Pcna-positive cardiomyocytes in *cpt1b*^OE^ embryos by 18% relative to vehicle treatment (Figure 3E), demonstrating that inhibiting Glul can effectively mitigate the cardiomyocyte proliferation induced by *cpt1b* overexpression. Taken together, these data suggest that Glul may play a pivotal role in modulating the effects of *cpt1b* on cardiomyocyte proliferation, potentially uncovering a novel metabolic regulatory axis in cardiac development.

## 4. Discussion

The present study demonstrates that *cpt1b* plays a critical role in regulating cardiomyocyte proliferation during zebrafish heart development. Our findings show that loss of *cpt1b* function leads to impaired cardiomyocyte proliferation, whereas cardiomyocyte-specific overexpression of *cpt1b* promotes cardiomyocyte proliferation. Notably, we identified glutamine synthetase (Glul) as a key downstream effector of *cpt1b* in regulating cardiomyocyte proliferation.

Cardiomyocyte proliferation is essential for heart development and regeneration after injury. Numerous studies have demonstrated that metabolic pathways are involved in the regulation of cell proliferation in the context of heart regeneration [6,7,37,38,39]. Nevertheless, the explorations of their roles in early cardiac development remain relatively scarce. The present study offers new insights into the role of Cpt1b in early cardiac development and extends its function in regulating cellular proliferation, although additional proliferation assays at later timepoints are necessary to fully understand the influence of Cpt1b on cardiomyocyte proliferation. Consistent with our findings, a recent study discovered that fatty acid oxidation and *cpt1b* are vital for cardiomyocyte proliferation during zebrafish heart regeneration, which involved NF-κB signaling and increased inflammatory response [20]. Conversely, other studies show that inhibition of fatty acid oxidation and/or Cpt1b can enhance cardiomyocyte proliferation and heart regeneration in mice [18,19]. Adding to the complexity, while mouse *Cpt1b* knockout results in lethality [40], our findings, along with another study [21], demonstrate that zebrafish *cpt1b* mutants are viable. The disparities in cardiomyocyte proliferation and organismal viability between zebrafish and mice suggest that the function of Cpt1b and/or fatty acid oxidation in cardiomyocyte proliferation may be intricate and species- and/or context-dependent.

The catabolism of fatty acids and glutamine generates substrates that converge on the Krebs cycle at disparate points, indicating potential interaction and synergy between these two metabolic pathways in the regulation of tissue growth and homeostasis [41,42,43,44]. Indeed, CPT1 and glutaminase (GLS)-mediated Angiopoietin-like protein (ANGPTL4)-induced energy metabolism and cell proliferation in non-small cell lung cancer (NSCLC) cells [45], whereas the simultaneous inhibition of CPT1 and GLS enhanced the suppression of proliferation and migration of CB-839-resistant breast cancer cells [46]. In *Cpt1b*-deficient mouse cardiomyocytes, a substantial increase in α-ketoglutarate, an intermediate in glutamine catabolism, was observed, whereas the levels of glutamine and glutamate were not affected [19]. Our RNA sequencing analysis suggests that *cpt1b* can regulate glutamine biosynthesis by modulating glutamine synthetase expression, implying a potential direct interaction between fatty acid oxidation and glutamine synthesis that extends beyond the confines of the Krebs cycle. Notwithstanding this finding, further investigations are necessary to elucidate the precise details and underlying mechanisms of the direct interaction between fatty acid and glutamine metabolism.

In conclusion, this study uncovers a novel mechanism whereby *cpt1b* facilitates zebrafish cardiomyocyte proliferation through the modulation of *glul* expression, which sheds new light on the intricate metabolic regulation of cardiac development, homeostasis, and regeneration and provides potential therapeutic targets for the development of innovative, metabolism-based interventions for heart diseases.

## Data Availability

The raw sequence data generated in this study have been deposited to the Genome Sequence Archive [47] in China National Center for Bioinformation under the accession number CRA019362.

## Supplementary Materials

The following supporting information can be downloaded at: https://www.mdpi.com/article/10.3390/jcdd11110344/s1, Table S1: Results of differentially expressed gene analysis of *cpt1b* knockout and overexpressing hearts compared to their wild-type siblings.
